# Systemic Bioinformatic Analyses of Nuclear-Encoded Mitochondrial Genes in Hypertrophic Cardiomyopathy

**DOI:** 10.3389/fgene.2021.670787

**Published:** 2021-05-12

**Authors:** Zhaochong Tan, Limeng Wu, Yan Fang, Pingshan Chen, Rong Wan, Yang Shen, Jianping Hu, Zhenhong Jiang, Kui Hong

**Affiliations:** ^1^Department of Cardiovascular Medicine, The Second Affiliated Hospital of Nanchang University, Nanchang, China; ^2^Department of Science and Technology, The Second Affiliated Hospital of Nanchang University, Nanchang, China; ^3^Jiangxi Key Laboratory of Molecular Medicine, The Second Affiliated Hospital of Nanchang University, Nanchang, China

**Keywords:** hypertrophic cardiomyopathy, microarrays, bioinformatics analysis, nuclear-encoded mitochondrial genes, transcription factors

## Abstract

Hypertrophic cardiomyopathy (HCM) is an autosomal dominant disease and mitochondria plays a key role in the progression in HCM. Here, we analyzed the expression pattern of nuclear-encoded mitochondrial genes (NMGenes) in HCM and found that the expression of NMGenes was significantly changed. A total of 316 differentially expressed NMGenes (DE-NMGenes) were identified. Pathway enrichment analyses showed that energy metabolism-related pathways such as “pyruvate metabolism” and “fatty acid degradation” were dysregulated, which highlighted the importance of energy metabolism in HCM. Next, we constructed a protein-protein interaction network based on 316 DE-NMGenes and identified thirteen hubs. Then, a total of 17 TFs (transcription factors) were predicted to potentially regulate the expression of 316 DE-NMGenes according to iRegulon, among which 8 TFs were already found involved in pathological hypertrophy. The remaining TFs (like GATA1, GATA5, and NFYA) were good candidates for further experimental verification. Finally, a mouse model of transverse aortic constriction (TAC) was established to validate the genes and results showed that DDIT4, TKT, CLIC1, DDOST, and SNCA were all upregulated in TAC mice. The present study represents the first effort to evaluate the global expression pattern of NMGenes in HCM and provides innovative insight into the molecular mechanism of HCM.

## Introduction

Hypertrophic cardiomyopathy (HCM) is an autosomal dominant genetic disease that is mainly characterized by ventricular hypertrophy with asymptomatic or serious complications such as sudden cardiac death (SCD), heart failure, and thrombosis (Marian and Braunwald, 2017). The prevalence of HCM in the general population was estimated to be 1/500 (Gersh et al., 2011), which was underestimated due to the limited HCM diagnostic technology. HCM is considered a leading cause of SCD in younger people and the leading cause of heart failure in cardiac diseases originating primarily from the myocardium (Weissler-Snir et al., 2019).

Normal myocardial energy metabolism from mitochondria is also an important material basis for keeping the normal heart tissue structure and the internal environment stable. Cardiac function will inevitably be impaired by mitochondrial dysfunction. Clinical and experimental studies have shown that the myocardial energy source switching from fatty acid oxidation to glycolysis is a common event in HCM (Tian, 2003). Mutations in a wide spectrum of nuclear-encoded mitochondrial genes (NMGenes) have been reported to be able to cause HCM characterized by impaired mitochondrial function (Marin-Garcia and Goldenthal, 2002b). For example, mutations in *ELAC2* (*ElaC* ribonuclease *Z 2*) encoding a short form of RNase Z were found to be associated with HCM (Saoura et al., 2019). Mitochondrial function depends on proteins encoded by both mitochondrial DNA (mtDNA) and nuclear DNA (nDNA). The mitochondrial proteome has been estimated to contain approximately 1000–1500 proteins, more than 99% of which are encoded by nuclear DNA (nDNA), while mtDNA refers to only 13 protein-coding genes (Pfanner et al., 2019). Considering the importance of mitochondria in HCM and the fact that functional proteins in mitochondria are encoded mainly by nDNA genes, exploring the function of NMGenes in HCM would help us better understand the novel role of mitochondria in the development of HCM.

With the development of genetic studies, high-throughput omics technologies (such as DNA microarrays and next-generation sequencing) that investigate gene function and expression at the genome-wide level have been widely used in basic research, clinical diagnosis, drug research and other fields. As a powerful technique, gene expression microarray-based bioinformatics analyses have also been widely used to identify HCM-related genes or noncoding RNAs, possible molecular mechanisms, and biological pathways (Lim et al., 2001; Yang et al., 2015; Hu et al., 2019; Li et al., 2019; Liu et al., 2019). For example, microarray analysis was performed to explore the expression pattern of lncRNAs (long noncoding RNAs) and mRNAs (messenger RNAs) in HCM, which identified hundreds of differentially expressed lncRNAs and genes (Yang et al., 2015). A recent study systemically analyzed RNA-seq data from 28 HCM patients and 9 healthy controls and identified 43 potential pathogenic variants in 19 genes and four subnetworks with significant roles in the progression of HCM (Gao et al., 2020). Although previous studies have highlighted the importance of integrative gene expression analysis in exploring the molecular mechanism of HCM, a systemic analysis of the expression pattern of NMGenes in HCM patients has never been reported.

To investigate the potential role of NMGenes in the pathogenesis of HCM, in this study, we performed a computational systems biology analysis based on large-scale HCM-related transcriptional data. A total of 316 differentially expressed NMGenes (DE-NMGenes) were identified. Based on these DE-NMGenes, gene ontology (GO) and pathway enrichment analyses were performed, and 17 KEGG-dysregulated pathways were identified. We also constructed a PPI (protein-protein interaction) network that consisted of 215 DE-NMGenes and 440 interactions. Finally, a total of 17 TFs (transcription factors) were predicted to potentially regulate the expression of the 316 DE-NMGenes. We provided a systematic view of the roles of mitochondrial genes in HCM and revealed some available candidates for future experimental verification.

## Results

### Nuclear-Encoded Mitochondrial Genes Are Significantly Changed in HCM

**FIGURE 1:**
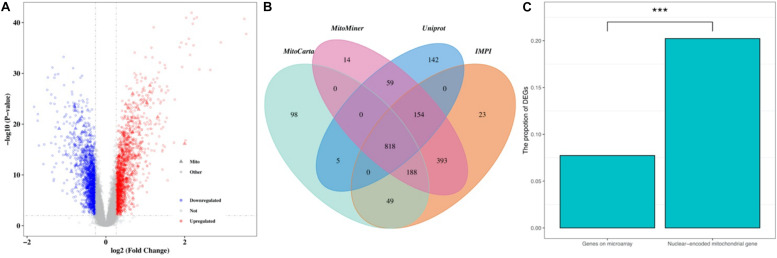
The expression pattern of NMGenes in HCM. **(A)** A volcano plot shows DEGs in HCM detected from GSE36961. The vertical lines correspond to 1.2-fold up and down, respectively, and the horizontal line represents a BH-corrected *p* value of 0.05. Therefore, red and blue dots represent upregulated and downregulated DEGs, respectively. Triangular nodes and circle nodes represent NMGenes and the other genes, respectively. **(B)** A four-way Venn diagram shows the number of NMGenes collected from the MitoCarta, MitoMiner, IMPI, and UniProt databases. **(C)** A bar plot shows the proportion of DEGs in all genes on microarray and NMGenes. The percentage of DEGs in NMGenes was significantly larger than the percentage of DEGs in overall genes detected on microarray (Fisher’s exact test, *p*-value < 2.20 × 10^–16^). *** represents *P*-value < 0.001.

The normalized gene expression dataset GSE36961 was downloaded from the GEO (Gene Expression Omnibus) database^1^, which included 107 HCM samples and 40 control samples (Clough and Barrett, 2016). Differentially expressed genes (DEGs) between HCM and the corresponding control samples were detected using the “Limma” package from R software (Ritchie et al., 2015). By keeping genes with a BH (Benjamini-Hochberg)-corrected *p*-value less than 0.01 and fold change (FC) larger than 1.2, we obtained 2927 DEGs, 1499 of which were upregulated and the remaining 1428 were downregulated (Figure 1A and Supplementary Table 1). To explore the expression pattern of NMGenes in HCM, we collected 1943 mitochondrial genes from the MitoCarta (Calvo et al., 2016), MitoMiner (Smith and Robinson, 2019), IMPI and UniProt databases (UniProt, 2019) (Figure 1B, see section “Materials and Methods” for details). After removing 13 mtDNA-encoded genes, 1930 NMGenes were retained for further analysis. Among these genes, 1562 genes were detected on microarray, and 316 genes were differentially expressed. Compared with the overall genes detected on the microarray, the proportion of DEGs in NMGenes was significantly higher (Figure 1C, Fisher’s exact test, *p*-value < 2.20 × 10^–16^). The extensive expression changes of NMGenes in HCM indicate that mitochondria play critical roles in the progression of HCM. Table 1 lists the top ten upregulated and downregulated NMGenes in HCM. Among these genes, four upregulated genes [namely, *PDK4* (*thpyruvate dehydrogenase kinase isozyme 4*), *STAT3* (*Signal Transducer and Activator of Transcription 3*), *HCLS1* (*Hematopoietic Cell-Specific Lyn Substrate 1*) and *FKBP11* (*FKBP Prolyl Isomerase 11*)], and four downregulated genes [namely, *GATM* (*Glycine Amidinotransferase*), *ATPIF1* (*ATP Synthase Inhibitory Factor Subunit 1*), *CPT1B* (*Carnitine Palmitoyltransferase 1B*), and *GJA1* (*Gap Junction Protein Alpha 1*)] have already been proven to play important roles in pathological hypertrophy (summarized in Table 1).

**TABLE 1:** Top 10 upregulated and top 10 downregulated DE-NMGenes.

Gene symbol	logFC	*P*-value	Roles in pathological hypertrophy
***PDK4***	2	6.79E-17	ANG II induced cardiac hypertrophy was associated with a marked upregulation of *PDK4* (Mori et al., 2012)
*DDIT4* (DNA-damage-inducible transcript 4)	1.32	3.94E-22	–
***STAT3***	1.12	2.38E-28	Pharmacologic inhibition of *STAT3* with WP1066 could suppress Ang II-induced myocyte hypertrophy (Chen et al., 2017).
***HCLS1***	1.12	4.52E-27	Changes in phospholipid metabolism occur in mammalian hypertrophied myocardium (Reibel et al., 1986).
*TKT* (Transketolase)	1.04	6.31E-25	–
*CLIC1* (Chloride Intracellular Channel 1)	0.88	6.85E-16	–
*ACTB* (Actin Beta)	0.87	4.59E-17	–
*DDOST* (Dolichyl-Diphosphooligosaccharide—Diphosphooligosaccharide-Protein Glycosyltransferase Non-Catalytic Subunit)	0.86	7.14E-21	–
***FKBP11***	0.85	1.11E-18	*FKBP11* was strongly and acutely induced in cardiac hypertrophy induced by TAC (Wang et al., 2019).
*TUBB* (Tubulin Beta Class I)	0.83	1.26E-23	–
***GATM***	−1.19	7.50E-20	In *GATM*-deficient mouses, hypertrophic marker *NPPA* expression was significantly upregulated (Jensen et al., 2020).
*SNCA* (Synuclein Alpha)	−1.01	8.68E-15	–
*CASQ1* (Calsequestrin 1)	−0.87	2.36E-11	–
*LYPLAL1* (Lysophospholipase Like 1)	−0.8	2.01E-24	–
***ATPIF1***	−0.79	4.15E-24	The knockout of *ATPIF1* protected the heart from myocardial hypertrophy induced by transverse aortic constriction or isoproterenol infusion (Yang et al., 2017).
*SDSL* (Serine Dehydratase Like)	−0.78	2.14E-23	–
*KLHDC9* (Kelch Domain Containing 9)	−0.77	7.07E-20	–
*DPYSL4* (Dihydropyrimidinase Like 4)	−0.76	2.80E-08	–
***CPT1B***	−0.75	1.07E-14	*CPT1B* deficiency could cause lipotoxicity in the heart under pathological stress, leading to exacerbated cardiac pathology (He et al., 2012).
***GJA1***	−0.75	1.28E-12	In HCM patients with valvular aortic stenosis, compensated hypertrophy had increased levels and increased lateral *CJA1* expression (Fontes et al., 2012).

*Genes with verified roles in pathological hypertrophy are marked in bold.*

### Downregulated DE-NMGenes Are More Functionally Diverse Than Upregulated DE-NMGenes

**FIGURE 2:**
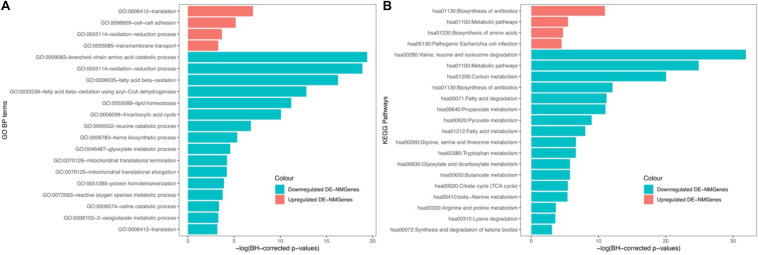
GO and KEGG enrichment results of 141 upregulated DE-NMGenes and 175 downregulated DE-NMGenes. Bar graph of the significantly enriched BP **(A)** and KEGG **(B)**. Blue and red bars represent the enriched BP terms or KEGG pathways for upregulated and downregulated DE-NMGenes, respectively.

GO biological process (BP) and KEGG pathway enrichment analyses for 316 DE-NMGenes were performed using DAVID (Database for Annotation, Visualization, and Integrated Discovery) (da Huang et al., 2009). Although the numbers of upregulated and downregulated DE-NMGenes were similar, downregulated DE-NMGenes were more functionally diverse than upregulated DE-NMGenes. By keeping terms with BH-corrected *p*-values less than 0.05, we obtained 4 GO BP terms and 4 KEGG pathways for 141 upregulated DE-NMGenes and 16 GO BP terms and 17 KEGG pathways for 175 downregulated DE-NMGenes (Figures 2A,B and Supplementary Table 2). The top 3 enriched GO terms in downregulated DE-NMGenes were “oxidation-reduction process,” “branched-chain amino acid catabolic process” and “fatty acid beta-oxidation.” The GO terms “oxidation-reduction process” and “translation” were both enriched in 141 upregulated and 175 downregulated DE-NMGenes. KEGG pathway enrichment analysis showed that downregulated DE-NMGenes were significantly enriched in energy metabolism-related pathways such as “Carbon metabolism” (15 genes, BH-corrected *p*-value = 1.94^∗^10^–9^), “Pyruvate metabolism” (7 genes, BH-corrected *p*-value = 1.25^∗^10^–4^), “Fatty acid metabolism” (7 genes, BH-corrected *p*-value = 3.24^∗^10^–4^), and “Citrate cycle” (5 genes, BH-corrected *p*-value = 4.35^∗^10^–3^). For upregulated DE-NMGenes, the KEGG pathways “Biosynthesis of antibiotics” (15 genes, BH-corrected *p*-value = 1.75^∗^10^–5^) and “Biosynthesis of amino acids” (7 genes, BH-corrected *p*-value = 8.88^∗^10^–3^) were significantly enriched.

### A Group of 215 DE-NMGenes Are Biologically Connected to Form a Network

**FIGURE 3:**
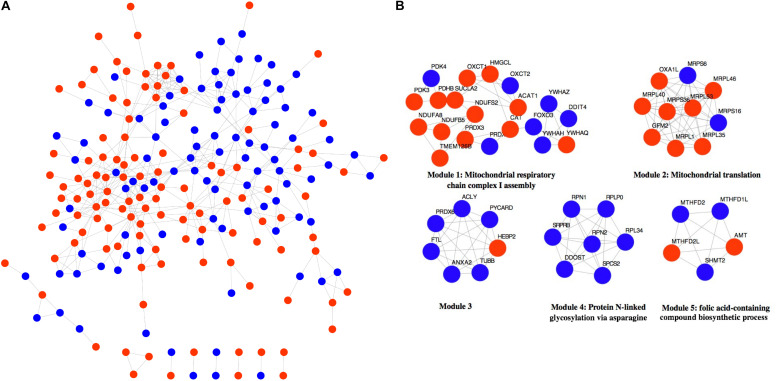
Network analysis of 316 DE-NMGenes. **(A)** The PPI network consists of 440 edges and 215 nodes. Red and blue nodes represent upregulated and downregulated DE-NMGenes, respectively. **(B)** The network representation of modules detected from the PPI network. Red and blue nodes represent upregulated and downregulated NMGenes, respectively.

The 316 DE-NMGenes were analyzed together to construct a PPI network. Consequently, a PPI network including 440 interactions and 215 nodes was obtained by using STRING (Search Tool for the Retrieval of Interacting Genes/proteins database) (Szklarczyk et al., 2019), with parameters including a minimum required interaction score larger than 0.7 (high confidence) and only query proteins being displayed. Thus, 215 out of the 316 DE-NMGenes were included in the final PPI network (Figure 3A). The 316 DE-NMGenes had significantly more interactions than would be expected (*p*-value < 2.2^∗^10^–16^) from a randomly chosen set of proteins of the same size drawn from the genome. In a PPI network, highly connected nodes are called hubs, which are expected to play an important role in understanding the biological mechanism of disease (Barabasi and Oltvai, 2004). Then, we calculated the degree for each node and selected genes with the degree ranked in the top 5% as hubs. Of the 215 nodes in the PPI network, 13 nodes were ranked in the top 5% and selected as hubs (Table 2). *DLD* (*dihydrolipoamide dehydrogenase*) was the hub gene with the largest degree and interacted with 19 proteins in the PPI network.

**TABLE 2:** Hubs in the PPI network.

Gene symbol	Full name	Degree
*DLD*	Dihydrolipoamide Dehydrogenase	19
*ACLY*	ATP Citrate Lyase	13
*CAT*	Catalase	13
*ACADM*	Acyl-CoA Dehydrogenase Medium Chain	12
*HADH*	Hydroxyacyl-CoA Dehydrogenase	12
*MRPL46*	Mitochondrial Ribosomal Protein L46	12
*MRPL53*	Mitochondrial Ribosomal Protein L53	11
*MRPL1*	Mitochondrial Ribosomal Protein L1	11
*MRPL40*	Mitochondrial Ribosomal Protein L40	11
*MRPS16*	Mitochondrial Ribosomal Protein S16	11
*ACAT1*	Acetyl-CoA Acetyltransferase 1	11
*RPLP0*	Ribosomal Protein Lateral Stalk Subunit P0	11
*OXA1L*	*OXA1L* Mitochondrial Inner Membrane Protein	11

**DLD*, patients with point mutations (p.D479V and p.R482G) at the *DLD* homodimer interface were affected with HCM (Shany et al., 1999; Odievre et al., 2005). *CAT, JMJD1A* (Jumonji domain containing 1A) represses the development of cardiomyocyte hypertrophy by upregulating the expression of *CAT* (Zang et al., 2020). *ACLY*, *ACLY* was associated with TAC (thoracic aortic constriction) induced cardiac hypertrophy by regulating autophagy (Marino et al., 2014).*

The MCODE (Molecular Complex Detection) plugin in Cytoscape was used to detect network modules from the PPI network (Bader and Hogue, 2003). A module is a group of closely related proteins that act in concert to perform specific biological functions through a PPI network that occurs in time and space (Lin et al., 2015). A total of 12 modules were extracted from the PPI network, of which five modules (modules 1–5) had nodes ≥ 5 (Figure 3B and Supplementary Table 3). The associated BPs for module 1, module 2, module 4 and module 5 were “mitochondrial respiratory chain complex I assembly,” “mitochondrial translation,” “protein N-linked glycosylation via asparagine” and “folic acid-containing compound biosynthetic process,” respectively, but no-GO term was significantly enriched in module 3.

### TFs Potentially Regulating DE-NMGenes Play Key Roles in HCM

iRegulon (Janky et al., 2014), available as a Cytoscape plugin, was used to predict TFs potentially regulating the 316 DE-NMGenes. A total of 17 TFs were obtained with the minimum normalized enrichment score >3 and the FDR on motif similarity <0.001 (Table 3). The TF with the largest number of targets is *PBX3* (*pre-B-*cell leukemia transcription factor 3), which regulates 145 DE-NMGenes. Eight of the 17 TFs, namely, *BACH1* (*BTB Domain and CNC Homolog 1*), ATF3 (*Activating Transcription Factor 3*), *XBP1* (*X-Box Binding Protein 1*), *KLF4* (*Kruppel Like Factor 4*), *MEF2C* (*Myocyte Enhancer Factor 2C*), *JUND* (*JunD Proto-Oncogene*), *MYC (MYC Proto-Oncogene*) and *YY1* (*YY1 Transcription Factor*), have already been proven to play important roles in pathological hypertrophy. The remaining nine genes with unknown roles in HCM were good candidates for further experimental verification.

**TABLE 3:** TFs potentially regulating the expression of 316 DE-NMGenes.

#TF	NES	#Targets	Function in pathological hypertrophy
*ATF4* (Activating Transcription Factor 4)	6.73	21	–
***BACH1***	5.27	101	Deletion of *BACH1* caused significant reductions in left ventricular hypertrophy (Mito et al., 2008).
*NFYA* (Nuclear Transcription Factor Y Subunit Alpha)	4.77	75	–
*PBX3* (PBX Homeobox 3)	4.45	145	–
*NFYC* (Nuclear Transcription Factor Y Subunit Gamma)	4.34	117	–
***ATF3***	4.32	46	Ectopic expression of *ATF3* was sufficient to promote cardiac hypertrophy (Koren et al., 2013).
***XBP1***	3.98	43	Myocardial XBP1s protein was significantly increased in hypertrophic and failing heart (Duan et al., 2016).
***KLF4***	3.63	45	Overexpression of *KLF4* in neonatal rat ventricular myocytes inhibits cardiomyocyte hypertrophy (Liao et al., 2010).
***MEF2C***	3.54	91	*MEF2C* silencing attenuated load-induced left ventricular hypertrophy by modulating mTOR/S6K pathway in mice (Pereira et al., 2009).
*GATA1* (GATA Binding Protein 1)	3.37	19	–
***JUND***	3.27	15	*JUND* could attenuate phenylephrine-mediated cardiomyocyte hypertrophy by negatively regulating AP-1 transcriptional activity (Hilfiker-Kleiner et al., 2006).
*IRF2* (Interferon Regulatory Factor 2)	3.27	34	–
*MYBL2* (MYB Proto-Oncogene Like 2)	3.27	90	–
***MYC***	3.17	17	*MYC* overexpression could induce cardiac hypertrophy (Olson et al., 2013).
*GATA5* (GATA Binding Protein 5)	3.14	13	–
*RARA* (Retinoic Acid Receptor Alpha)	3.12	9	–
***YY1***	3.02	39	*YY1* could prevent cardiac hypertrophy (Sucharov et al., 2008)and suppresses dilated cardiomyopathy and cardiac fibrosis (Tan et al., 2019).

*#TFs regulating DE-NMGenes are showed. TFs already proved to play important roles in pathological hypertrophy are marked in bold.*

### Validation of the Differentially Expressed NMGenes *in vivo*

**FIGURE 4:**
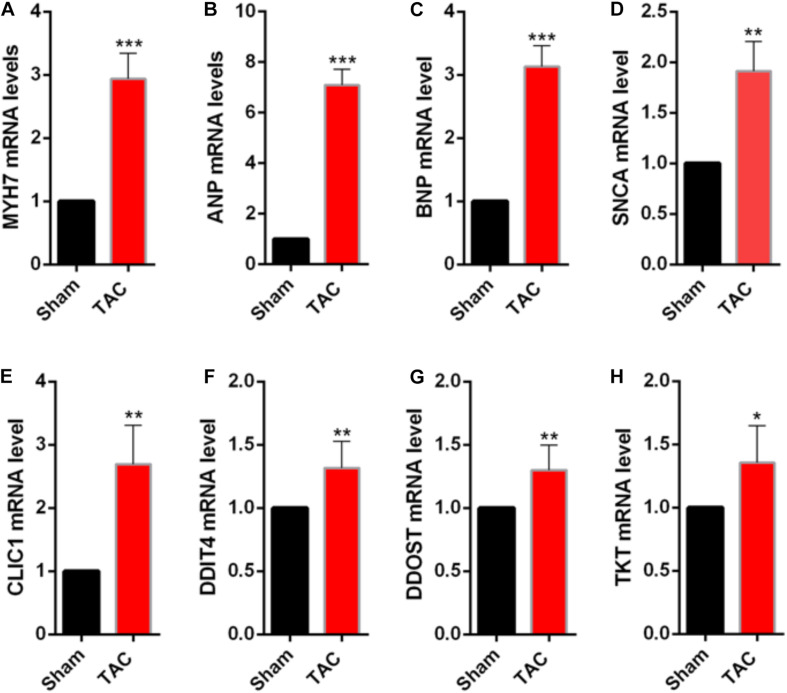
Validation of the differentially expressed NMGenes in TAC mice. **(A–C)** mRNA of the genes indicating cardiac hypertrophy increased. **(D–H)** mRNA of the differentially expressed NMGenes also increased. Data are presented as the mean ± SD. *P < 0.05, **P < 0.01, ***P < 0.001 (Student’s *t*-test); *n* = 5 samples per group. TAC, transverse aortic constriction.

To validate the identified genes *in vivo*, the samples were extracted from control and transverse aortic constriction (TAC) mice to identify whether the mRNA levels of the top five genes that have not been proven to play important roles in cardiac hypertrophy were consistent with the bioinformatic analysis. In the TAC group, *MYH7*, *ANP*, and *BNP* expression levels were increased (Figures 4A–C), indicating that pressure overload successfully induced cardiac hypertrophy in the mouse TAC model. Interestingly, the expression of *DDIT4, TKT, CLIC1, DDOST*, and *SNCA* in the mouse TAC model were all increased compared with the sham operation group (Figures 4D–H).

## Discussion

Although many studies have been conducted to explore the pathogenesis of HCM, the role of mitochondria in HCM development and progression remains largely unknown. More than 99% of mitochondrial proteins are encoded by nDNA, so NMGenes are more responsible for mitochondrial function (Ferramosca and Zara, 2013). Exploring the expression pattern of NMGenes in HCM will help us better understand the molecular mechanism of mitochondria in HCM. Therefore, we performed a comprehensive comparative analysis of NMGenes in HCM by comparing transcriptional data in HCM patients and normal healthy controls.

Differential expression analysis showed that the proportion of differentially expressed genes in NMGenes was significantly higher than the proportion of overall genes detected on the microarray, highlighting the importance of NMGenes in HCM. For the top 10 NMGenes with the highest fold change, four upregulated genes (i.e., *PDK4*, *STAT3*, *HCLS1*, and *FKBP11*) and four downregulated genes (i.e., *GATM*, *ATPIF1*, *CPT1B*, and *GJA1*) have already been shown to play important roles in pathological hypertrophy (Table 1). Importantly, the other genes with undetermined roles in HCM are good candidates for further experimental verification. Consistent with the bioinformatic analysis, *DDIT4, TKT, CLIC1* and *DDOST* mRNA expression increased in TAC mice compared with the sham operation group, suggesting that these genes may play an important role in promoting pathological hypertrophy. However, in contrast with the bioinformatic analysis, its expression at the mRNA level increased at 4 weeks after TAC. We speculate that it would decrease in the earlier or later time of TAC, as the duration of the pressure overload can affect the expression of associated genes (Souders et al., 2012). These DE-NMGenes provide a new perspective on the mechanisms in HCM. For example, *CLIC1*, as a metamorphic protein, is abundantly expressed in the heart (Ponnalagu et al., 2016); however, its function in the heart is far from fully understood. Direct evidence has shown that *CLIC1* plays a significant role in ischemia-reperfusion (IR) injury by regulating reactive oxygen species (ROS) generation (Gururaja Rao et al., 2020). Previous studies have demonstrated that abnormal production of ROS in cardiomyocytes is closely related to the occurrence and development of HCM (Hafstad et al., 2013; Brown and Griendling, 2015). We speculate that *CLIC1* is involved in the progression of HCM by regulating the generation of ROS and might be a potential therapeutic target for cardiac hypertrophy. *DDIT4* is an inhibitor of mTOR signaling, which plays a key regulatory role in cardiovascular pathology (Sciarretta et al., 2014). It is possible that *DDIT4* is involved in the progression of HCM by regulating mTOR signaling.

KEGG pathway analysis showed that abnormal expression of metabolically related pathways such as pyruvate metabolism and fatty acid metabolism (Figure 2B) may contribute to the pathogenesis of HCM. In the normal heart, mitochondrial fatty acid oxidation is the main (70–80%) source of energy, and the remaining 20–30% of ATP production derives largely from glucose oxidation (Sacchetto et al., 2019). Fatty acid metabolism disturbances are common in HCM patients, and mutations in the fatty acid oxidation pathway can result in HCM (Marin-Garcia and Goldenthal, 2002a). Fatty acid oxidation involves two key steps: fatty acid transfer and ββ-oxidation. Our results showed that genes involved in fatty acid transport (*CPT1B and CPT2)* or β-oxidation (*ACADSB, ACADM, ACADL*, and *HADH)* were all significantly downregulated (Supplementary Table 1). *CPT1B* was one of the top 10 downregulated DE-NMGenes and its deficiency could cause heart lipotoxicity, leading to exacerbated cardiac pathology (He et al., 2012). *ACADM* and *HADH* were two hubs in the PPI network. Rats with hypertrophic myocardium showed impaired fatty acid oxidation and decreased expression of *ACADM* and *ACADL* (Doenst et al., 2010). In rats with cardiac hypertrophy caused by left ventricular volume overload, HADH activity was significantly reduced (Lachance et al., 2014). Pyruvate metabolism is a key step in glucose oxidation. Compared with normal hearts, glucose oxidation was actually lower in hypertrophied hearts (Allard et al., 1997). Glucose oxidation and fatty acid oxidation are under fine regulation during disease progression, although there is still controversy, allowing us to consider the treatment of HCM from the perspective of energy metabolism. New treatments include inhibiting enzymes related to fatty acid oxidation and directly increasing the oxidation of glucose and pyruvate, which may bring light to patients with cardiomyopathy in the future.

Genes rarely act alone and usually perform their functions in connection with other genes. Moreover, genes with relatively small but significant changes in expression can also contribute to the phenotypes of interest. However, differential expression analysis focuses only on individual gene expression without considering its close connection with other genes. PPI network-based analysis might largely overcome these limitations by combining gene expression and connections. In the present study, we performed PPI network analysis and obtained 440 interactions among 215 DE-NMGenes. We found that compared with random gene sets from the genome, DE-NMGenes formed significantly more interactions, which indicated that DE-NMGenes were biologically connected to form a group. Our results identified five closely connected modules that might contribute to the development of HCM. In addition, we highlighted 13 hub genes with a high level of network connectivity but relatively modest changes in expression. Hubs *DLD, CAT, ACADM*, and *HADH* have already been proven to be involved in the progression of HCM or pathological hypertrophy, and the role of the remaining hubs in HCM deserves further investigation. The top 2 hub gene, *ACLY*, is an essential cytosolic enzyme for generating acetyl-CoA, a key metabolite for glucose, fatty acid, and amino acid catabolism. A mendelian randomization study found *ACLY* to be a promising target for cardiovascular protection (Ference et al., 2019). In addition, we also noticed that five of the 13 hubs were genes encoding mitochondrial ribosomal proteins (MRPs), which assist protein synthesis within mitochondria. These MRPs were grouped into module 2 in the network module analysis (Figure 3B). Mutations in *MRPL3*, *MRPS14*, *MRPS22*, and *MRPL44* could cause HCM accompanied by multiorgan diseases (Smits et al., 2011; El-Hattab and Scaglia, 2016). Therefore, these MRPs are functionally connected, and the consistent downregulation of *MRPL46*, *MRPL53*, *MRPL1*, and *MRPL40* may cause mitochondrial translation deficiency, which would result in a severe phenotype in HCM.

Generally, gene expression is under the fine turn regulation of TFs. Among the 17 TFs predicted in this work, more than half have been shown to be associated with pathological hypertrophy (Table 3). The remaining 9 TFs (i.e., *ATF4*, *NFYA*, *PBX3*, *NFYC*, *GATA1*, *MYBL2*, *GATA5*, and *RARA* were good candidates for further experimental verification. *NFY* (*nuclear transcription factor Y*) is a heterotrimeric TF complex consisting of three subunits, *NFYA*, *NFYB* and *NFYC*. In this work, *NFYA* and *NFYC* were predicted to regulate 75 and 117 DE-NMGenes, respectively. By analyzing the targets of *NFYA* and *NFYC* in DE-NMGenes, we found that they were both enriched in the GO term “negative regulation of apoptotic process” with *p*-values of 4.1^∗^10^–7^ and 2.5^∗^10^–4^, respectively. Although the role of *NFY* in cardiovascular disease has not been reported, *NFY* is involved in cancer by regulating apoptosis (Gurtner et al., 2010). Moreover, *NFYA* and *NFYC* were both significantly differentially expressed in HCM. We speculate that *NFYA* and *NFYC* may be involved in the pathogenesis of HCM by regulating apoptosis, which provides us with a new perspective to understand the relationship between *NFY* and HCM.

The GATA TF family comprises six members (named GATA1-6) that are involved in the regulation of growth, differentiation, survival and maintenance of body function. Previous studies have underscored the pivotal roles of the GATA family in cardiac hypertrophy (Pikkarainen et al., 2004). Mutations in *GATA2*, *GATA4*, and *GATA6* were identified in patients with HCM (Alonso-Montes et al., 2017). Overexpression of either *GATA4* or *GATA6* could induce cardiac hypertrophy both *in vitro* and *in vivo* (Liang et al., 2001). *GATA5* and *GATA1* are closely related to cardiomyopathy diseases such as dilated cardiomyopathy, although their role in HCM has not yet been reported (Zhang et al., 2015). In this work, *GATA1* and *GATA5* were predicted to regulate 19 and 13 DE-NMGenes, respectively (Table 3). Given that the functional characteristics of *GATA5* and *GATA1* overlap at least partly with those of other *GATA* TFs and that *GATA1* and *GATA5* regulate DE-NMGenes, it is reasonable to speculate that *GATA1* and *GATA5* may contribute to HCM.

## Conclusion

The present study was the first effort to evaluate the global expression pattern of NMGenes in HCM. Based on differential expression analysis, we found that NMGenes were significantly changed and identified 316 DE-NMGenes. Further GO enrichment analysis showed that downregulated DE-NMGenes were more functionally diverse. These DE-NMGenes participated in 10 significant pathways, and nine of these pathways were metabolically related. PPI network analysis showed that 13 DE-NMGenes with high node connectivity were selected as hubs. Finally, a total of 17 TFs were predicted to potentially regulate the expression of the 316 DE-NMGenes, and TFs (such as *ATF4, NFYA*, *NFYC, GATA1*, and *GATA5*) might play roles in HCM. This analysis will provide valuable information for future research on the molecular mechanisms of HCM and offer clues for the discovery of novel therapeutic strategies.

## Materials and Methods

### Data Collection

Normalized gene expression data (GSE36961) were collected from the GEO database (Clough and Barrett, 2016). NMGenes were collected from the MitoCarta (Version 2.0) (Calvo et al., 2016), MitoMiner (Version 4.0) (Smith and Robinson, 2019), IMPI^2^ and UniProt databases (UniProt, 2019).

### Differential Expression Analysis

To identify DEGs between HCM and normal healthy hearts, limma (Version 3.40.6), an R package in Bioconductor, was utilized (Ritchie et al., 2015). Genes with BH-corrected *p*-values less than 0.01 and fold changes (FCs) larger than 1.2 were selected as significantly differentially expressed. We have deposited the analysis code to a public repository^3^.

### Functional Enrichment Analysis

GO BP and KEGG pathway enrichment analyses of DE-NMGenes were performed using DAVID, an online functional annotation tool, to understand the biological significance of a list of genes (da Huang et al., 2009). In this work, GO BP and KEGG pathways with BH-corrected *p*-values less than 0.05 were selected as significant.

### PPI Network Construction

The PPI network of DE-NMGenes was constructed using the STRING database, and an online database provides information regarding the predicted and experimental protein interactions (Szklarczyk et al., 2019). In this work, PPIs between DE-NMGenes with interaction scores larger than 0.7 were retained.

### Network Module Analysis

A network module is defined as a group of genes participating in the same biological function. In this work, we detected network modules from the constructed PPI network using MCODE (Bader and Hogue, 2003), a plugin in Cytoscape^4^. Given the following parameters: a degree cutoff = 2, node score cutoff = 0.2, k-score = 2 and max. depth = 100, modules with scores > 3 and a number of nodes > 5 were selected. GO BP enrichment analysis of modules was performed using DAVID, and BH-corrected *p* values < 0.05 were selected as significant.

### Prediction of TFs Regulating DE-NMGenes

To predict TFs regulating DE-NMGenes, iRegulon (Version: 1.3), a plugin in Cytoscape, was employed (Janky et al., 2014). The iRegulon plugin uses motif and track discovery in a set of coregulated genes to identify regulons. Given the following parameters: motif collection (10 kb, 9,713 PWMs), track collection (1120 ChIP-seq tracks of ENCODE raw signals), putative regulatory region (20 kb centered around TSS), motif rankings database (20 kb region centered around TSS, 7 species), track of rankings database (20 kb centered around TSS, ChIP-seq-derived), minimum identity between orthologous genes = 0 and maximum false discovery rate on motif similarity = 0.001, TFs with the NES (normalized enrichment score) larger than 3 were selected. The higher the NES was, the more reliable the TFs were.

### Animals and Surgical Procedures

All experiments involving animals were approved by the Animal Ethics and Experimentation Committee of Nanchang University and carried out according to the “Guide for the Care and Use of Laboratory Animals.” Male C57BL/6 mice, aged 8 weeks and weighing 20–25g, were purchased from the SlacJingda Experimental Animals Company [Changsha, Hunan Province, China]. A total of 20 mice were divided into two groups (ten mice per group): the sham operation group and the TAC group. TAC was performed as previously described (Zhang et al., 2020). Briefly, mice were induced with 5% isoflurane and intubated orally and then maintained at 2% isoflurane during surgery with mechanical ventilation. After a midline sternotomy, the aortic arch was exposed. Constriction was performed by tying a 5-0 silk suture around a 27-gauge needle overlying the arch between the origin of the brachiocephalic trunk and left common carotid artery. For the sham operation group, 10 mice underwent the same procedure, but the suture was withdrawn without tying. Then, the thorax and skin were closed by using 6-0 polypropylene sutures. Four weeks after surgery, the mice were euthanized, and their hearts were quickly excised for further evaluation.

### Quantitative Real-Time PCR Analysis

Total RNA was extracted from mouse cardiac tissues using TRIzol reagent (Invitrogen, New York, United States), and then the quality and concentration of RNA were determined using an Agilent Bioanalyzer 2100 according to the manufacturer’s instructions. The cDNAs were generated by MMLV transcriptase (BioRAD, United States), and quantitative real-time PCR assays were performed as previously described (Yu et al., 2018). Triplicate PCR amplifications were performed for each sample, and the mRNA levels were normalized to GAPDH. The comparative threshold cycle method (2-ΔΔCT) was applied to estimate the relative gene expression of cardiac tissues between the TAC and sham operation groups. The primer sequences for *CLIC1, DDIT4, TKT, DDOST, SNCA, MYH7, ANP*, and *BNP* are listed in Supplementary Table 4. The differences in mRNA levels between the two groups were evaluated by using Student’s *t*-tests. A *P*-value < 0.05 was considered significant.

## Data Availability Statement

Publicly available datasets were analyzed in this study. This data can be found here: GEO (Gene Expression Omnibus) database, Accession number GSE36961: https://www.ncbi.nlm.nih.gov/geo/query/acc.cgi?acc=GSE36961.

## Ethics Statement

The animal study was reviewed and approved by the Animal Ethics and Experimentation Committee of Nanchang University.

## Author Contributions

KH and ZJ were responsible for the entire project and revised the draft of the manuscript. ZT, LW, and ZJ collected the data, performed the analyses, and drafted the first version of the manuscript. All authors took part in the interpretation of the results and preparation of the final version of the manuscript.

## Conflict of Interest

The authors declare that the research was conducted in the absence of any commercial or financial relationships that could be construed as a potential conflict of interest.

## Footnotes

1 https://www.ncbi.nlm.nih.gov/geo/

2 http://impi.mrc-mbu.cam.ac.uk/

3 https://github.com/ZhenhongJiang/Nuclear-encoded-mitochondrial-genes-in-HCM.git

4 https://cytoscape.org/

## Supplementary Material

Supplementary Table 1 2927 DEGs between HCM and control samples

Supplementary Table 2 Annotation results for 141 upregulated DE-NMGenes, 175 DE-NMGenes, and 4 network modules

Supplementary Table 3 Modules detected from PPI network

Supplementary Table 4 Real-time PCR Primer sequences.
