# Supplementary material for: Systemic Bioinformatic Analyses of Nuclear-Encoded Mitochondrial Genes in Hypertrophic Cardiomyopathy
Source: Front Genet. 2021 May 12;12:670787. doi: 10.3389/fgene.2021.670787 (PMC8150003; doi:10.3389/fgene.2021.670787)
Supplement: Supplementary Table 4 — Real-time PCR Primer sequences. [file Table_4.DOCX]

**Table S4.** Real-time PCR Primer Sequences

| **Target gene** | **Sequences (5’-3’)** |
| --- | --- |
| CLIC1 | forword: 5-GAAGAACAACCTCAGGTCGAAC -3  reverse: 5- CTCTGTCCGTCTCTTGGTGTC -3 |
| DDIT4 | forword: 5-CAAGGCAAGAGCTGCCATAG -3  reverse: 5-CCGGTACTTAGCGTCAGGG -3 |
| TKT | forword: 5-ATGGAAGGTTACCATAAGCCAGA -3  reverse: 5-TGCAGCATGATGTGGGGTG -3 |
| DDOST | forword: 5- GCTGGACAACCTGAACGTG -3  reverse: 5- TCCACCGACGGGGAAAAGA -3 |
| SNCA | forword: 5- CTTCCCTCTAATGCGCTGGTT -3  reverse: 5- CGTTGCGTTTCTTTTGATGTCA -3 |
| MYH7 | forword: 5-ACTGTCAACACTAAGAGGGTCA -3  reverse: 5-TTGGATGATTTGATCTTCCAGGG -3 |
| ANP | forword: 5- GCTTCCAGGCCATATTGGAG -3  reverse: 5- GGGGGCATGACCTCATCTT -3 |
| BNP | forword: 5- GAGGTCACTCCTATCCTCTGG -3  reverse: 5- GCCATTTCCTCCGACTTTTCTC -3 |
| GAPDH | forword: 5- AGGTCGGTGTGAACGGATTTG -3  reverse: 5-TGTAGACCATGTAGTTGAGGTCA -3 |
